# The Influence of 5′,8-Cyclo-2′-Deoxyguanosine on ds-DNA Charge Transfer Depends on Its Diastereomeric Form: A Theoretical Study

**DOI:** 10.3390/antiox12040881

**Published:** 2023-04-04

**Authors:** Bolesław T. Karwowski

**Affiliations:** DNA Damage Laboratory of Food Science Department, Faculty of Pharmacy, Medical University of Lodz, ul. Muszynskiego 1, 90-151 Lodz, Poland; boleslaw.karwowski@umed.lodz.pl

**Keywords:** (5′*R*/*S*) 5′,8-cyclo-2′-deoxyguanosine, 8-OXO-dG, clustered DNA damage, DNA repair, charge transfer, density functional theory

## Abstract

The genetic information stored in the nucleobase sequence is continuously exposed to harmful extra- and intra-cellular factors, which can lead to different types of DNA damage, with more than 70 lesion types identified so far. In this article, the influence of a multi-damage site containing (5′*R*/*S*) 5′,8-cyclo-2′-deoxyguanosine (cdG) and 7,8-dihydro-8-oxo-2′-deoxyguanosine (^OXO^dG) on charge transfer through ds-DNA was taken into consideration. The spatial geometries of oligo-RcdG: d[A_1_(5′*R*)cG_2_A_3_^OXO^G_4_A_5_]*d[T_5_C_4_T_3_C_2_T_1_] and oligo-ScdG: d[A_1_(5′*S*)cG_2_A_3_^OXO^G_4_A_5_]*d[T_5_C_4_T_3_C_2_T_1_] were optimized at the M06-2X/6-D95**//M06-2X/sto-3G level of theory in the aqueous phase using ONIOM methodology. For all the electronic property energies under discussion, the M06-2X/6-31++G** level of theory was used. Additionally, the non-equilibrated and equilibrated solvent-solute interactions were into consideration. The obtained results confirm the predisposition of ^OXO^dG to radical cation formation regardless of the presence of other lesions in a ds-DNA structure. In the case of electron transfer, however, the situation is different. An excess electron migration towards (5′*S*)cdG was found to be preferred in the case of oligo-ScdG, while in the case of oligo-RcdG, ^OXO^dG was favored. The above observation was confirmed by the charge transfer rate constant, vertical/adiabatic ionization potential, and electron affinity energy values, as well as the charge and spin distribution analysis. The obtained results indicate that 5′,8-cyclo-2′-deoxyguanosine, depending on the C5′ atom chirality, can significantly influence the charge migration process through the double helix. The above can be manifested by the slowdown of DNA lesion recognition and removal processes, which can increase the probability of mutagenesis and subsequent pathological processes. With regard to anticancer therapy (radio/chemo), the presence of (5′S)cdG in the structure of formed clustered DNA damage can lead to improvements in cancer treatment.

## 1. Introduction

Each day in a single human cell, 10^5^ DNA lesions are formed, whether single, tandem, or clustered (CDL) lesions. These are all the result of extra- or intra-cellular factors such as ionization radiation (IR), xenobiotics, and reactive oxygen/nitrogen species (ROS/NOS) [1]. The appearance of the lesion within genetic information can cause mutation (transversion or transition) and in turn lead to cancer, aging, or evolution acceleration [2]. On the other hand, their formation in the genome of a cancerous cell can be perceived as the basis of effective radio/chemo or combined anticancer treatment [3,4]. The ROS and NOS can be generated in the cell environment, e.g., by water molecule radiolysis, or they can be the product of specific reactions such as Fenton or Haber-Weiss.

Moreover, approximately 85% of oxygen is converted to water in mitochondria. During a four-electron reduction process, different species can be formed. Of these, H_2_O_2_ (molecular species) seems to be less reactive than other free radicals such as the superoxide radical (O_2_^●−^), and hydroxyl radical (HO●) [5]. The level of ROS and NOS in a cell is controlled by several specific enzymes which constitute the first line of cellular defense against their unwanted activity, e.g., catalase (CAT), superoxide dismutase (SOD), glutathione peroxidase, etc. [6,7]. The HO● is the most powerful ROS which can react with different macromolecules with diffusion-controlled rate constants of 10^10^ Lmol^−1^s^−1^. The hydroxyl radical can interact with nucleosides in one of two manners: (a) by hydrogen atom abstraction from a sugar moiety (15%) or (b) by HO● addition to an unsaturated bond of nucleo-base (85%) [8]. The rate of hydrogen atom abstraction from a sugar moiety depends on its position and was found as follows, in [%]: H5′/5′′(57) > H4′(22) > H3′(17) > H2′/2′′(13) > H1′(11) [9]. The above corresponds well with the steric hindrance and solvent-accessible surface area in the DNA double helix [10]. If this process occurs in the oligonucleotide structure, it can lead to different consequences such as phosphodiester or glycosidic bond disruption. Additionally, in a hypoxic condition, the abstraction of one of the hydrogen atoms from the C5′ function with subsequent C5′-C8 molecular cyclization can lead to (5′*R*/*S*)5′,8-cyclo-2′-deoxyguanosine (cdG) formation [11]. However, the cdG level depends on the source of the tissue, isolation, and measurement methods. In a recent study, their frequency was measured in untreated human breast adenocarcinoma cells (MCF-7) by LC-MS/MS techniques as 0.05 and 0.11 per 10^6^ nucleosides of 5′*R* and 5′*S*cdG respectively [12]. On the other hand, the HO● addition to the purine bases was noted mainly at the C4, C5, and C8 atoms with rate constants *k* ≥ 10^9^ M^−1^s^−1^ and a yield at a rate of 50%, 13%, and 37% respectively [13]. The addition to the C4 atom of dG leads to radical cation or neutral radical formation, which can be subsequently converted to imidazoline or oxazolone [14,15]. Conversely, the formation of an 8-OH-adduct radical of guanine with further one-electron oxidation leads to 7,8-dihydro-2′-deoxyguanosine (^OXO^dG). ^OXO^dG, thanks to its abundance (14 per 10^6^ bases), stability, and relatively easy measurement, is often used as a marker of several pathologic processes [16,17]. The above is a direct result of the fact that 2′-deoxyguanosine shows the lowest ionization potential (*E*^O^_dG/dG•_ = 1.29 V) of all nucleosides, and because of that is sensitive to different harmful oxidizing agents [18].

DNA lesions formed in the genome form the substrates for several specific DNA repair systems such as base/nucleotide excision repair (BER, NER), homologous recombination (HR), non-homologous end joining (NHEJ), etc. [19,20]. The lesions discussed above, i.e., ^OXO^dG and cdG, are removed by different machinery, i.e., BER and NER respectively. However, if the repair systems are defective, an unrepaired lesion can result in mutation, carcinogenesis, aging, neurodegenerative disorders, etc. [21]. On the other hand, if cdG is not removed from the genome, as in the case of *xeroderma pigmentosum* (a defect in NER), it can influence the repair process of a second lesion, e.g., ^OXO^dG [22,23,24,25]. By way of example, 7,8-dihydro-8-oxo-guanine can form a noncanonical pair with adenine (^OXO^G::A), which, if not repaired, gives rise to G→T transversion. Adenine from ^OXO^G: A is removed by a specific glycosylase MutY [26]. Barton et al. postulated that the above protein can scan the genome toward the DNA damage site by electron transfer between two proteins containing [4Fe-4S] clusters [27]. It has been assumed by Lin et al. that ^OXO^G: A can accept the ejected electron and trigger MutY into action [28]. At the beginning of the 21st century, it was discovered that not only glycosylases but also other proteins like helicases and polymerases contained [4Fe-4S] clusters [29]. This suggests that the protein in question can interact/communicate by charge transfer through a double helix. Though the charge transfers through ds-DNA has been investigated for the last three decades, for the purpose of this study, mainly canonical oligonucleotides were examined. Several experimental techniques have been applied to resolve the nature of the discussed process, including electron paramagnetic resonance, voltammetry, atomic force microscopy, and/or scanning tunneling microscopy [30,31,32,33]. Even though the methodologies are well-known, their usefulness with regard to DNA damage has some limitations depending on lesion stability, synthetic problems, and incorporation into the investigated oligonucleotide. Because of this, the value of theoretical methods like Density Functional Theory (DFT) is inestimable in these fields. Furthermore, a computational approach gives unlimited access for investigating charge transfer, depending on the context of the base sequences, the form of the double helix (A, B, or Z), high-level structures (tetraplex, triplex, Holliday junction), etc. Because of the costly and time-consuming nature of classical experimental techniques, the influence of the base sequence on charge transfer was determined by systematic study. Additionally, the formation of DNA damage in the genome depends on whether the form of DNA is single or double-stranded, as has been observed in the case of 5-carboxamido-5-formamido-2-iminohydantoin, 5-guanidinohydantoin, (5′*R*) and (5′*S*) 5′,8-cyclo-2′-deoxypurines, etc. [34]. It should be pointed out that the abundance/predisposition of DNA lesions depends on the condition of the cellular environment (e.g., hypoxic or normoxic), which was especially visible in the case of 2,6-diamino-4-hydroxy-5-formamidopyrimidine; and 7,8-dihydro-8-oxo-2′-deoxyguanosine [35]. Taking all the above into consideration, a theoretical approach for charge transfer does look tempting. However, even though new functional achieve high accuracy as well as increased calculation power, some limitations should be pointed out: firstly, the complicated nature of the system (ds-DNA) allowed only short fragment optimization in the condensed phase; secondly, the quantum molecular dynamics with a high-level basis set containing a dispersion and polarization function is possible only for simple systems like a base/nucleoside pair or their dimer [36]. Because of this, for an extended system, a vertical rather than adiabatic approach was applied. Though this process has been well investigated in the case of native base pairs, little is known about the influence of DNA lesions other than ^OXO^G on charge transfer through the double helix.

Because of the nature of charge migration through the double helix, in these studies, short ds-DNA in its neutral, cation, and anion forms was taken into consideration. The above strategy allowed investigation of the “global” structural and electronic changes of the discussed ds-oligo forced by electron adoption or loss. Additionally, the proposed ds-oligo contained a clustered damage part consisting of 5′,8-cyclo-2′-deoxypurine, and 7,8-dihydro-8-oxo-2′deoxyguanosine. The above allowed a direct comparison of the electronic properties of the mentioned lesions.

With this in mind, this article represents the first theoretical investigation into the influence of both cdG diastereomers, i.e., 5′*R* and 5′*S,* together with ^OXO^dG on electron and hole migration through double-stranded DNA.

## 2. Materials and Methods

### 2.1. Computation Methodology of ONIOM Studies

**Figure 1:**
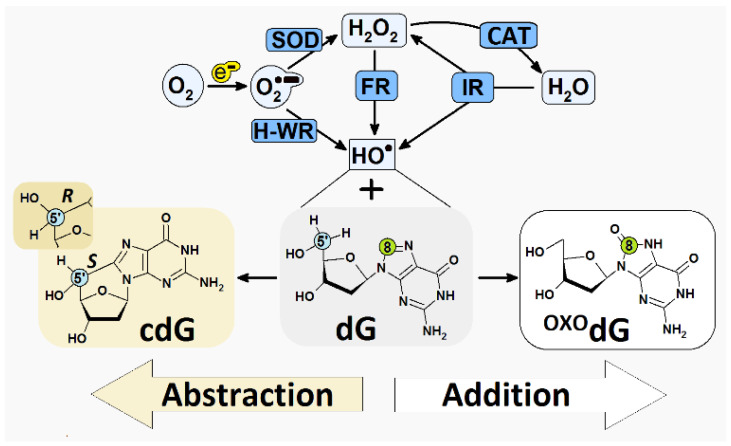
Graphical representation of hydroxyl radical formation by a Fenton type reaction (FR), Haber-Weiss Reaction catalyzed by transition metal ions (H-WR) and by ionization radiation activity (IR) and product of HO● reaction with dG. SOD-superoxide dismutase, CAT- catalase, ^OXO^dG-7,8-dihydro-2′-deoxyGuanosine, cdG-5′,8-cyclo-2′-deoxyguanosine.

The structure of the discussed ds-oligos in their neutral, positively (cation), and negatively (anion) charged forms was optimized at the M062x/D95** level of theory in the aqueous phase and presented in Figure 1 Oligo-RcdG: d[A_1_(5′R)cG_2_A_3_^OXO^G_4_A_5_]*d[T_5_C_4_T_3_C_2_T_1_] and oligo-ScdG: d[A_1_(5′S)cG_2_A_3_^OXO^G_4_A_5_]*d[T_5_C_4_T_3_C_2_T_1_] calculations were performed using ONIOM (our Own N-layered Integrated Molecular Orbital and Molecular Mechanics) methodology [37]. For these calculations, because of the complexity of the system, the structures of the ds-pentamers in question were divided into two layers: high (nucleobases), M06-2X/D95**, and low (sugar-phosphate backbone) M06-2X/sto-3G levels of theory in the aqueous phase [38]. Following Leszczynski and Schaefer’s previous studies, the negative charges of all the phosphate groups were neutralized by the addition of a proton, instead of counterions [39,40,41]. This significantly reduced calculation costs. The strategy has been well documented as applicable to charge/proton transfer or structural studies of nucleic acids, as well as being adopted for low electron migration between base aromatic rings and a sugar-phosphate backbone. The obtained ds-pentamers were subsequently converted into a base-pair ladder, which was further used for electronic property energy calculations. Because only heterocycle rings are involved in charge transfer, the sugar-phosphate backbone was removed from the obtained structures, with subsequent atom saturation with the necessary hydrogen atoms, which were subsequently optimized at the M06-2X/D95** level of theory in the aqueous phase.

### 2.2. Computation Methodology of DFT Study

All energy calculations were performed in the aqueous phase by density functional theory (DFT) methodology using the M06-2X functional with an augmented polarized valence double-ζ basis set 6-31++G** [38,42]. The M06-2X functional and 6-31++G** basis set was used for all calculations because of their relative cost-effectiveness and efficiency, as outlined in previous studies [43]. The transition dipole moment of excited states and the single point calculation at the M06-2X/6-31++G** level of theory were performed using the time-dependent DFT (TD-DFT) method [44,45]. The solvent effect was described for an aqueous medium, applying Tomasi’s polarized continuum model (PCM) [46]. The solvent effect was looked at in two modes, i.e., non-equilibrium (NE) and equilibrated (EQ), following a previously described methodology) [47]. The energy of molecules in the non-equilibrated solvent-solute interaction was calculated using two-step processes according to the save-read procedures implemented in the Gaussian G16 software package. For all optimized structures, a charge and spin analysis was achieved using the Hirshfeld theory [48]. The electron coupling was calculated according to the Generalized Mulliken–Hush methodology [49]. The electronic properties, i.e., adiabatic ionization potential (AIP), adiabatic electron affinity (AEA), vertical ionization potential (VIP), and vertical electron affinity (VEA), were calculated as previously described [50]. In brief, the following energy notation was used: the *E*_geometry_^charge^ of the molecule (neutral form) is described as *E***_0_^0^**, the vertical cation/anion as *E***_0_^+^**/*E***_0_**, the adiabatic cation/anion as *E***_+_^+^**/_+_*E***_-_^−^**, and the vertical neutral formed from a cation/anion state as *E***_0_^+^**/*E***_0_^−^**. The difference, given in eV, between the mentioned energies corresponds to the suitable electronic states and is described as follows: VIP^NE^ = *E*_0_^+(NE)^ − *E*_0_^0^ (vertical ionization potential in the NE state); VIP^EQ^ = *E*_0_^+(EQ)^ − *E*_0_^0^ (vertical ionization potential in the EQ state); AIP = *E*_+_^+^ − *E*_0_^0^ (adiabatic ionization potential); VEA^NE^ = *E*_0_^–(NE)^ − *E*_0_^0^ (vertical electron affinity in the NE state); VEA^EQ^ = *E*_0_^–(EQ)^ − *E*_0_^0^ (vertical electron affinity in the EQ state); AEA = *E*_0_^0^ − *E*_—_(adiabatic electron affinity).

All calculations were performed in the aqueous phase on the Gaussian G16 (version C.01) software package [51]. The RMSD values were calculated using BioVia Discovery Studio v20.1.0.19295 software [52]. The three-dimensional structural analyses of the mentioned ds-DNAs, based on a standard reference frame, were obtained by a 3DNA software package using the web-based interface w3DNA (web 3DNA) [53].

## 3. Results and Discussion

Both the discussed lesions cdG and ^OXO^dG possess mutagenic potential if not properly repaired. As mentioned above, ^OXO^dG can be removed from the genome by BER, while (5′*R/S*)cdG is removed exclusively by NER. The mutagenic potential of (5′*S*)cdG was noted at a rate of 34% in *Escherichia coli* and the cd G→A, cdG→T, and dC deletions were observed. In addition, it has been shown that (5′*S*)cdG is removed two times more slowly by UvrABC than by (5′*R*)cdG [54]. Because of this, the possible accumulation of cdG in the genome can lead to local multi-damage site formation with other more abundant lesions like ^OXO^dG.

**Figure 2:**
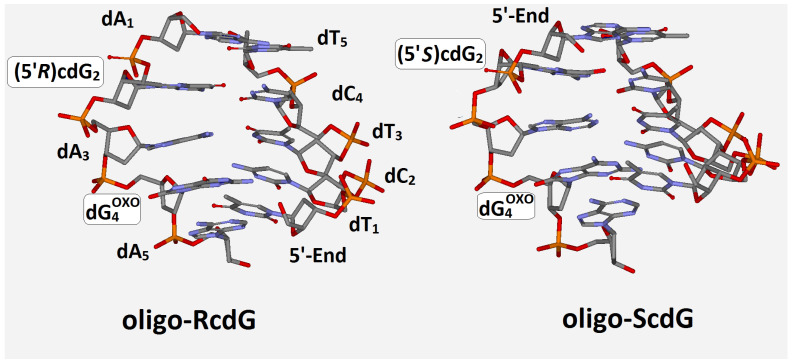
Graphical representation of oligo-RcdG and oligo-ScdG optimized structures at the M06-2x/D95** level of theory in the aqueous phase.

Here, the double-stranded (*ds*-) oligonucleotides containing cdG and ^OXO^dG as model clustered DNA damage noted as oligo-RcdG: d[A(5′*R*)cGA^OXO^GA]*[TCTCT] and oligo-ScdG: d[A(5′*S*)cGA^OXO^GA]*[TCTCT] were taken into theoretical consideration (Figure 2).

### 3.1. The Influence of (5′R/S)cdG on the Double Helix Structure in Comparison with ^OXO^dG

5′,8-cyclo-2′-deoxyguanosine belongs to a unique class of DNA lesion known as a tandem lesion, in which both the sugar and base moieties have been damaged by a single ionization event. The additional covalent bond between C5′ and C8 atoms inhibits rotation around the glycosidic bond C1′-N9 and C4′-C5′ making its structure extremely rigid. As a result, modified nucleosides adopt an *anti*-conformation, while the 5′OH group is frozen at *gauche (-)* or *trans* orientation in the 5′*S* and 5′*R* diastereomers of cdG respectively. Conversely, the flexibility of 7,8-dihydro-8-oxo-2′-deoxyguanosine was found to be unrestricted, which allows ^OXO^dG to adopt a *syn*-conformation and form a non-canonical pair with dA which is prohibited in the case of both cdG diastereometric forms. A structural analysis of oligo-RcdG and oligo-ScdG was performed according to the standard reference frame for the description of nucleic acid base pair geometry [55,56]. Additionally, for further discussion, a structural analysis was performed for the discussed ds-DNA in neutral and positively or negatively charged forms. Of the many complementary base-pair and base-pair step parameters, rise, tilt, the overlap of base-pair ring atoms only as well as the distance between purine C8 and pyrimidine C6 functions were chosen as valuable for a discussion on charge transfer through a double helix structure (Table 1).

Additionally, the Root-Mean-Square Deviation (RMSD) values of atomic positions for oligo-RcdG and oligo-ScdG in their Neutral, Anionic, and Cationic forms were also calculated [57]. An analysis of the above-mentioned factors, in particular RMSD, showed that electron loss by ds-oligo induced more notable structural changes than electron adoption. Comparing the cationic versus neutral and anionic versus neutral forms of the spatial geometries of oligo-RcdG and oligo-ScdG gave the following RMSD values of ds-DNA: 0.54/0.66 and 0.22/0.36 [Å^2^], respectively (Table 1). Moreover, in the cases of both ds-oligos, the sugar-phosphate frame was far more flexible than the internal base-pair ladders. Additionally, it should be noted that at each point of the RMSD analysis, oligo-ScdG had a higher geometry susceptibility than oligo-RcdG towards electron loss or adoption. The above results are coincidental with the overlap of the base-pair ring (BPO), which showed that in the radical cation form, the BPO between cG_2_C_4_ and A_3_T_3_ BPs decreases by up to 0.75/0.83 [Å^2^] while in the case of A_3_T_3_|^O^G_4_C_2_it increases by 0.8/0.72 [Å^2^] for oligo-RcdG/oligo-ScdG, respectively. In the case of the remaining BP dimers, this parameter was found as almost unchanged, as shown in Table 1. Surprisingly, the excess-electron appearing in both ds-oligo structures (adiabatic anion forms) did not affect the base-pair overlapping within base-pair dimers. The difference between the BPO of a neutral form of ds-oligo and the BPO of its anionic form adopts a value in the range of 0.01–0.26 [Å^2^], as shown in Table 1.

The discussed overlap of the base-pair ring is well related to the *Rise* parameter, which describes the distance between two neighboring base pairs within a double helix. As was expected, the electron loss by ds-DNA induced visible *Rise* factor increases between the cG_2_C_4_ and A_3_T_3_ base pairs by 0.13/0.04 [Å] with subsequent distance shortening in the case of the A_3_T_3_|^O^G_4_C_2_ dimer by 0.34/0.24 [Å] for oligo-RcdG/oligo-ScdG, respectively. A different pattern was noted after adiabatic anion formation by the discussed ds-DNA. The excess electron adoption by oligo-RcdG leads to distance increases within the [cG_2_A_3_]*[T_3_C_4_] and [A_3_^O^G_4_]*[C_2_T_3_] BP-dimer of 0.11 [Å] and 0.15 [Å], respectively. While the *Rise* parameter assigned for the corresponding dimers isolated from oligo-ScdG shows more significant increases in both cases, i.e., 0.3 [Å] of the [cG_2_A_3_]*[T_3_C_4_] and 0.24 [Å] of [A_3_^O^G_4_]*[C_2_T_3_] systems (Table 1). Taking all the above into consideration, it can be postulated that in both ds-oligo cases, the ^O^G_4_C_2_ base-pair should be privileged to radical cation formation/adoption, while the excess electron should settle at the ^O^G_4_C_2_ base-pair of oligo-RcdG and at cG_2_C_4_ of oligo-ScdG. Additionally, differences in the influence of the cdG5′*R* and 5′*S* diastereomers on the structure of the double helix were well visible in the case of the *Tilt* parameter calculated for the [A_1_cG_2_]*[C_4_T_5_] moiety. In all the discussed ds-oligo forms (neutral, cationic, anionic) *Tilt* was noted at a level of 1.6 [^O^] and 11.4 [^O^] for oligo-RcdG and oligo-ScdG, respectively. Parallel to the above, the distance fluctuation between the complementary base atoms C8 (Purine) and C6 (Pyrimidine) was noted mostly at a level of 0.1 [Å] (Table 1). Exclusively, in the cases of A_3_T_5_ of oligo-RcdG, distance increases by 0.2 [Å] and decreases within ^O^G_4_C_2_ of oligo-ScdG by 0.2 [Å] were observed when the ds-oligo lost the electron. The above supports the previous prediction that a base pair containing ^OXO^G is predisposed to electron-hole adoption.

### 3.2. The Influence of (5′R/S)cdG on Charge and Spin Distribution within the Double Helix

**Figure 3:**
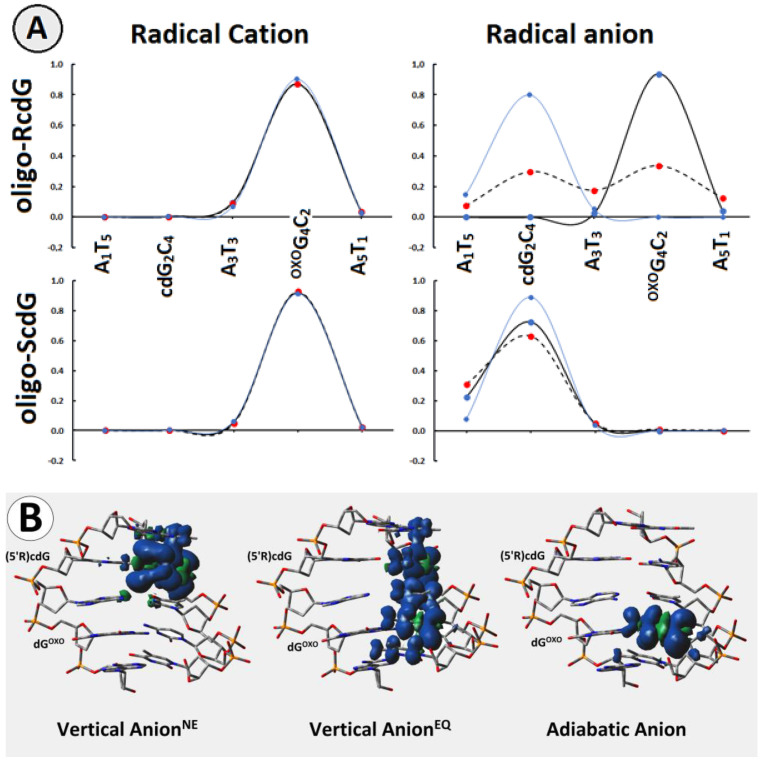
(**A**) Spin distribution within oligo-RcdG and oligo-ScdG calculated at the M062x/6-31++G** level of theory in the condensed phase, with the whole double helix taken into consideration. _‾‾‾_●_‾‾‾_ adiabatic radical cation/anion, _‾‾‾_●_‾‾‾_ vertical radical cation/anion, non-equilibrated solvent-solute interaction (NE), ---●--- vertical radical cation/anion, equilibrated solvent-solute interaction (EQ). The raw data of charge and spin distribution have been given in Supplementary Materials Table S1. (**B**) Graphical visualization of excess electron distribution within oligo-RcdA.

As has been discussed above, the clustered DNA lesion appearing in the double helix structure induces different structural changes depending on the form of the cdG diastereomeric subunit. Conversely, previous research results indicated that the second moiety, i.e., ^OXO^dG, causes only negligible disturbance in the geometry of ds-oligo. Additionally, most charge transfer studies are discussed at the level of canonical or lesioned ds-DNA in the vertical cation/anion mode. The adiabatic approach is considered less attractive as it is time-consuming (and has high calculation costs). In their recent article, Sevilla et al. examined solution-solute equilibrate and non-equilibrated interaction [47]. These strategies provide valuable information about the charge and spin distribution at the electron-loss or adoption point of induction within ds-DNA. Based on the previous conclusion that purines adopt a positive charge while pyrimidines a negative one, to simplify the charge migration analysis and to avoid an unexpected interaction, the investigated double helix contains exclusively purine and pyrimidine strands [58,59]. Depending on the spatial geometry of the double helix, an electron hole or excess-electron can migrate along the strands exploiting the nature of stacked bases, and forming a local anion or cation within the system. Therefore, the influence of CDL containing two different lesions, i.e., (5′*R*/*S*)cdG and ^OXO^dG, on charge and spin distribution are worth examining. It has been investigated according to Hirschfeld’s methodology [48]. Furthermore, in investigating this process, Mabesoone et al. considered the influence of non-equilibrated solvent-solute interaction [60]. The charge and spin distribution analysis within the double helix after electron loss and vertical cation formation were found to be coincident. As shown in Figure 3A, the electron hole was exclusively located at the ^OXO^G_2_C_4_ moiety in both investigated ds-oligos, regardless of the solution-solute interaction mode. None of the above was found on the base pairs containing (5′*R*) or (5′*S*)cdG.

Therefore, these results are in good agreement with the fact that 7,8-dihydro-8-oxo-2′-deoxyguanosine adopts the lowest ionization potential among all the nucleobases of the investigated systems (discussed below). It can thus be concluded that ^OXO^dG::C constitutes a significant radical cation sink in the vertical and adiabatic modes of the discussed process.

The situation becomes different when an excess electron appears in the ds-DNA structure. Firstly, the negative charge/spin was mainly (90%) located at the (5′*S*)cdG_2_C_4_ unit in the structure of oligo-ScdG, after nucleus relaxation, i.e., the adiabatic anion form. The remaining 10% was dispersed on the proximal A_1_T_5_ and A_3_T_3_ base pairs. However, the charge and spin distribution calculated at the vertical mode in non-equilibrated solvent-solute interaction was located at A_1_T_5_ (23%), cdG_2_C_4_ (73%), and A_3_T_3_ (4%). Surprisingly, after solution-solute interaction equilibration, the charge, and spin undergo a higher dispersion over the A_1_T_5_ base pair of up to 31%, with subsequent decreases on the cdG_2_C_4_ to 62%. The changes in the cdG diastereomer to 5′*R* (oligo-RcdG) shift the electron’s final destination point from cG_2_C_4_ to the ^OXO^G_4_C_2_ base pair, as previously observed in the oligonucleotide containing ^OXO^dG as an isolated lesion. However, the negative charge distribution analysis of oligo-RcdG vertical anion calculated in a non-equilibrated solvent–solute interaction mode showed that the (5′*R*)cdG_2_C_4_ base pair is predisposed to excess electron adoption at a rate of 80%, leaving the remaining 20% dispersed over proximal base-pairs. Solvent-solute equilibration interaction leads to excess electron propagation over all the base pairs of oligo-RcdG (7%-A_1_T_5_, 29%-(5′*R*)cdG_2_C_4_, 17%-A_3_T_3_, 33%-^OXO^dG_4_C_2_, 12%-A_5_T_1_), as shown in Figure 3A,B. The above leads to a highly effective “negative charge” transfer over a long distance and therefore allows glycosylases such as MutY, ExoIII to scan the genome in an extremely effective manner [26].

### 3.3. Electronic Properties of Oligo-RcdG and Oligo-ScdG

As shown in the previous section, the positive charge and corresponding spin are mainly located on purine moieties, while the negative charge is on pyrimidine [59]. This is in good agreement with previous studies [9]. However, their distribution strictly depends on the diastereomeric forms of cdG present in the oligo-RcdG or oligo-ScdG structure. It can therefore be expected that the above tandem lesion can infect the electronic properties of the remaining base pairs. Most of the available literature is theoretical and experimental studies of electronic properties concerning isolated systems (nucleic-base, nucleotides, nucleosides, or their pairs). Here, the base pairs extracted from ds-oligo are taken into consideration. The above allows the AIP, VIP, VEA, and AEA values to be obtained for the BPs in their geometry adopted in the neutral and charged double helix structure, at the M06-2x/6-31++G** level of theory in the aqueous phase. The electronic properties calculated of complete ds-oligos as well as the base-pair ladder did not indicate that the cdG diastereomeric form had a significant influence (see the data presented in Table 2).

However, equal decreases in the discussed parameters were observed after the removal of the sugar–phosphate backbone. Only in the case of vertical electron affinity calculated in the equilibrated solvent-solute interaction mode did the oligo-ScdG (−1.60 eV) show a higher predisposition to excess electron adoption than oligo-RcdG (−1.33 eV), which follows directly from the different spin distribution (Figure 3A). Due to the nature of double helix solvation and its dissimilarity to isolated base pairs, the above-mentioned electronic parameters were calculated and discussed in the equilibrated solvation-solute interaction mode. In all discussed cases, the lowest ionization potentials, vertical (5.93 eV) and adiabatic (5.56 eV), were found in the case of base pair containing 7,8-dihydro-8-oxo-2′-deoxyguanosine, i.e., ^OXO^G_4_::C_2_ (Table 2). Furthermore, no differences were noted between the source of the BP’s origin, oligo-RcdG or oligo-ScdG. Regardless of the discussed ds-oligo, the VIP and AIP of the cG_2_C_4_ moiety were noted, respectively, as 6.15 eV and 6.19 eV. For the remaining A_1_T_5_, A_3_T_3,_ and A_5_T_1_base pairs, the above parameters were in a range between 6.54 eV and 6.72 eV and were significantly higher than those found for cG_2_C_4_ and ^OXO^G_4_C_2_, which is in good agreement with the previous observation (Table 2 and Figure 3). The extra electron appearing in the system leads to vertical anion formation. A base-pair electronic properties analysis shows that in the case of oligo-RcdG, the highest vertical (−1.53 eV) and adiabatic (−1.93 eV) electron affinities were calculated for the ^OXO^G_2_C_4_ moiety. As expected, the VEA of G_2_C_4_, A_3_T_3,_ and A_5_T_1_ adopt similar values, in a range between −1.49 and −1.42 eV, which is well consistent with the previous spin analysis (Figure 3B). In the case of oligo-ScdG, the cG_2_C_4_ moiety showed a higher vertical and adiabatic electron affinity, i.e., −1.55 and −1.94 eV. Surprisingly, for this ds-oligo, the VEA calculated for the ^OXO^G_4_C_2_ base-pair was found to be at the same level as was found for oligo-RcdG. The above indicates that the predisposition of ^OXO^GC to excess electro-adoption depends on the other DNA lesions in its proximity

### 3.4. Charge Transfer through ds-DNA Containing (’’R/S)cdG and ^OXO^dG

As discussed previously, ds-DNA can be perceived as a nanowire [61]. The double-helix’s ability to charge transfer follows directly from the base-pairs’ capability of interacting together via stacking, while the sugar-phosphate backbone plays a negligible role in this process. Given the above, for these studies, only the internal BP ladder was taken into consideration. The excess electron or electron-hole migration process can be perceived in three categories: single-step tunneling, random-walk multistep, and polaron-like hopping [62,63]. The results of theoretical and experimental studies have shown that charge can migrate over a long distance—over 200 Å—within ds-DNA from the place of its induction thanks to an incoherent mechanism [64]. On the other hand, a single-step super exchange mechanism can be observed over a distance of a few base pairs. However, for all the above, the BP mutual positions and their electronic properties are essential for the charge transfer of ds-DNA in its oxidized or reductive state. The theory developed by Marcus takes all these discussed factors together in the equation of rate constant (k_ET_) presented below [65,66]. For further details, please refer to Voityuk’s review [67].

(1) $$k_{ET}=\frac{4\pi {\left|V_{da}\right|}^{2}}{h^{}}\sqrt{\frac{1}{4\pi \lambda k_{b}T}}\cdot \exp\left(\frac{-{\left(\Delta G+\lambda \right)}^{2}}{4\pi \lambda k_{b}T}\right)$$

k_b_—the Boltzmann constant, h—the Planck constant, T—temperature (K).

As shown, the charge transfer depends on the driving force Δ*G*, which is the free energy difference between the initial and final molecule states involved in charge transfer, while reorganization (λ) describes the energy changes of adjacent structures which occur during hole or electron migration. These two factors can be linked together in activation energy (*E*_a_), which depends on the intervening system. For these studies, only the process with negative Δ*G* was taken into consideration, as shown in Table 3. A higher absolute value of electron-hole migration driving force was found for A_3_T_3_→^OXO^G_4_C_4_ and ^OXO^G_4_C_4_←A_5_T_5_ in both ds-oligos at a level of close to 1 [eV]. While for A_1_T_1_→cG_2_C_2_ and cG_2_C_2_←A_3_T3, the value was noted as two times smaller, i.e., around 0.5 [eV]. For the discussed step, the *k*_ET_ was found in a range of 10^9^ to 10^12^ [s^−1^]. Surprisingly, negative values of λ and *E*_a_ were obtained in the case of electron hole transfer from the AT base-pair towards the cGC moiety (A_1_T_1_→cG_2_C_2_,cG_2_C_2_←A_3_T_3_), irrespective of the 5′8-cyclo-2′-deoxyguanosine diastereomeric form. These unusual observations (negative reorganization and activation energy) can be explained by the fact that the above parameters were calculated for the investigated base pair dimer isolated from the optimized ds-oligo. It should be pointed out here, that the spatial geometry of the base pair extracted from ds-DNA is different from that obtained for the isolated base pair (an ideal model).

The above means the simple base-pair extracted from ds-oligo, after charge changes, can adopt a geometry that is less privileged than that found for the previous stage. However, the global geometry of the ds-pentamer was in its ground state. The above was especially visible in the case of an AT crosslink; please see reference [68] for details. The observed negative λ and *E*_a_ values of the discussed process indicate that the hole transfer within the [A_1_T_1_]*[cG_2_C_2_] and [A_3_T_3_]*[cG_2_C_2_] base pair dimer should be extremely fast towards the cG_2_C_4_ moiety. On the other hand, an analysis of the Δ*G* values indicates that the radical cation migration from cG_2_C_2_ toward ^OXO^G_4_C_4_ transfer is privileged, i.e., *k*_ET_ = 10^13^ [s^−1^]. Additionally, the majority spin density was found on the ^OXO^G_4_C_2_ moiety of the ds-oligo in an adiabatic cation state. Based on the above and as ^OXO^G_4_C_2_ has the lowest ionization potential, this base pair can be assigned as the radical cation sink during electron hole transfer. This is in good agreement with previous observations [43,69,70]. No differences between oligo-RcdG and oligo-ScdG in the discussed values were observed.

Because of its nature, the charge transfer through ds-DNA may occur in its oxidative and reduced state. The transfer of excess electrons was found as privileged towards (5′*S*)cG_2_C_2_ in the case of oligo-ScdG, and towards ^OXO^G_4_C_2_ for oligo-RcdG. As shown in Table 3, the rate constant of A_1_T_1_→cG_2_C_2_ and cG_2_C_2_←A_3_T_3_ were as follows: 7.83 × 10^13^ s^−1^ and 4.34 × 10^13^ s^−1^ for oligo-ScdG; for oligo-RcdGit was calculated as 1.46 × 10^14^ s^−1^ and 1.2 × 10^14^ s^−1^ for A_1_T_1_→cG_2_C_2_ and cG_2_C_2_←A_3_T_3,_ respectively. Additionally, a different transfer predisposition between cG_2_C_4_ and ^OXO^G_4_C_2_ was observed. In the case of oligo-RcdG, it was cG_2_C_2_←^OXO^G_4_C_4_ (*k*_ET_ = 5.89 × 10^13^), while in the case of oligo-ScdG, it was noted as the opposite, i.e., cG_2_C_2_→^OXO^G_4_C_2_ (*k*_ET_ = 5.06 × 10^15^). The above is supported by results of adiabatic electron affinity and the negative charge and spin distribution calculation. It can thus be concluded that the presence of a different diastereomer (5′*S*) or (5′*R*) of 5,’8-cyclo-2′-deoxyguanosine can change the other DNA damage recognition processes by glycosylases (e.g., MutY, ExoIII) via an electron transfer process, which can ultimately give rise to an acceleration in the aging, carcinogenesis, and mutagenesis processes.

## 4. Conclusions

The genetic information stored in the nucleobase sequence is continuously exposed to harmful extra- and intra-cellular factors. Their activity can lead to different types of DNA damage, with more than 70 lesion types identified up to now. The stability of the genetic information is governed by DNA repair systems BER, NER, NHEJ, HR, etc. Most of the DNA damage is removed from ds-DNA by BER machinery. However, a lesion like 5′,8-cyclo-8-oxo-2′-deoxyguanosine (a tandem lesion) is the substrate of NER. Therefore, any defect in the NER system can have significant health implications. Moreover, the accumulation of cdG in the genome can cause multiple damage sites to form and subsequently affect further repairs to lesions. Recently, Barton et al. have shown that glycosylases like MutY can recognize a DNA lesion by charge transfer. Given the above, the influence of CDLs containing (5′*R*/*S*)cdG and ^OXO^dG on the above process was considered worth investigating. For this purpose, the following ds-oligs were chosen:

oligo-RcdG: d[A_1_(5′*R*)cG_2_A_3_^OXO^G_4_A_5_]*d[T_5_C_4_T_3_C_2_T_1_]

oligo-ScdG: d[A_1_(5′*S*)cG_2_A_3_^OXO^G_4_A_5_]*d[T_5_C_4_T_3_C_2_T_1_].

- A global structural analysis shows that oligo-ScdG containing (5′*S*)cdG had a greater geometry susceptibility than oligo-RcdG in which (5′*R*)cdG was present towards electron loss or adoption, which was shown by an RMSD value calculation.
- The electron loss causes *Rise* factor increases between cG_2_C_4_ and A_3_T_3_ base-pairs by 0.13/0.04 [Å] with subsequent distance shortening in the case of the A_3_T_3_; ^O^G_4_C_2_ dimer by 0.34/0.24 [Å] for oligo-RcdG/oligo-ScdG, respectively.
- The excess electron adoption by oligo-RcdG leads to *Rise* parameter increases within [cG_2_A_3_]*[C_4_T_3_] and [A_3_^OXO^G_4_]*[C_2_T_3_] by 0.11 [Å] and 0.15 [Å], respectively. Conversely, the *Rise* parameter assigned for the corresponding BP-dimers isolated from oligo-ScdG showed more significant increases in both cases, i.e., 0.3 [Å] for the [cG_2_A_3_]*[T_3_C_4_] and 0.24 [Å] for the [A_3_^O^G_4_]*[C_2_T_3_] systems.
- The charge and spin distribution analysis in vertical and adiabatic modes showed that the radical cation is mainly located at the ^OXO^dG_4_C_2_ base-pair, irrespective of the diastereomeric form of the second lesion. Contrary to the above, electron adoption by oligo-RcdG and oligo-ScdG leads to different consequences in the case of oligo-ScdG. The negative charge was mainly found at the (5′*S*)cdG_2_C_4_ base-pair (in vertical and adiabatic ds-oligo forms), while the presence of (5‘*R*)cdG leads to an initial negative charge appearing at (5′*R*)cdG_2_C_4_ with subsequent migration towards ^OXO^dG_4_C_2_.
- The charge transfer investigation, according to Marcus theory, revealed that in the case of (5′R/S)cdG, the radical cation migration towards the ^OXO^dG_4_C_2_ base pair is found to be privileged. However, in the case of excess electron transfer, differences were observed between (5′*R*) and (5′*S*)cdG when present in the ds-oligo structure. In the case of oligo-RcdG, the electron transfer towards the ^OXO^dG_4_C_2_ moiety was noted as privileged, while in the case of oligo-ScdG, it was privileged towards (5′*S*)cdG_2_C_4_.

Therefore, it can be concluded that in both ds-oligo cases, the ^OXO^G_4_C_2_ base-pair should be privileged to a suitable radical cation formation, while the excess electron should settle at ^OXO^G_4_C_2_ of oligo-RcdG and at cG_2_C_4_ of oligo-ScdG.

The above observation indicates that 5′,8-cyclo-2′-deoxyguanosine, depending on the C5′ atom chirality, can significantly affect the charge migration process through the double helix. This can be manifested by the slowdown of the DNA lesion recognition and removal process, which can increase the probability of mutagenesis and subsequent pathological processes. However, with regard to anticancer therapy (radio/chemo), the presence of (5′S)cdG in the structure of clustered DNA damage can result in improved cancer treatment.

## Figures and Tables

**Table 1:** Selected base-pairs and base-pair step parameters, as well as the overlap of base-pair ring atoms only (BPO). Root-Mean-Square Deviation (RMSD) of atomic positions calculated for oligo-RcdG and oligo- ScdG in their Neutral (N), Anionic (A), and Cationic (C) forms.

Base-Pair Dimer	Oligo-RcdG		
	BPO [Å^2^]	Rise [Å]	Tilt [^O^]
A_1_T_5_|cG_2_C_4_	3.90^N^/3.80^C^/3.89^A^	2.82/2.78/2.78	1.30/2.17/1.29
cG_2_C_4_|A_3_T_3_	3.55/2.80/3.54	2.97/3.10/3.08	−3.06/−0.6/−2.7
A_3_T_3_|^O^G_4_C_2_	3.71/4.51/3.73	3.29/2.95/3.44	1.56/1.62/−0.4
^O^G_4_C_2_|A_5_T_1_	4.04/3.96/4.03	3.15/3.01/3.10	−1.12/−0.5/−1.7
	oligo-ScdG		
A_1_T_5_|cG_2_C_4_	3.29/3.26/3.55	3.17/3.24/3.11	12.0/11.1/11.2
cG_2_C_4_|A_3_T_3_	4.21/3.38/4.12	2.90/2.94/3.20	−1.51/−3.6/−2.3
A_3_T_3_|^O^G_4_C_2_	3.79/4.15/3.91	3.25/3.01/3.01	−1.33/−1.2/−0.3
^O^G_4_C_2_|A_5_T_1_	4.19/4.08/4.16	3.16/3.02/3.31	−1.70/0.2/−1.7
Distance [Å]	Base-Pair	oligo-RcdG	oligo-ScdG
^Pu^ C8-C6^Py^	A_1_T_5_	9.8/9.8/9.8	9.7/9.8/9.8
	cG_2_C_4_	9.9/9.9/9.9	9.9/9.9/10
	A_3_T_3_	9.8/10/9.8	9.8/9.8/9.8
	^O^G_4_C_2_	9.9/9.9/10	10/9.8/9.9
	A_5_T_1_	9.8/9.8/9.8	9.8/9.7/9.7
RMSD [Å^2^]	Anion versus Neutral		
	ds-DNA	Base-Pair Ladder	Sugar-Phpsph. Frame
oligo-RcdG	0.22	0.20	0.24
oligo-ScdG	0.36	0.29	0.42
	Cation versus Neutral		
oligo-RcdG	0.54	0.44	0.62
oligo-ScdG	0.66	0.51	0.77

**Table 2:** Electronic properties, in [eV], of oligo-RcdG and oligo-ScdG as well as base-pairs isolated from the above ds-oligos: Vertical (VIP), Adiabatic Ionization Potential (AIP) and Vertical (VEA), Adiabatic (AEA) Electron Affinity, calculated at the M062x/6-31++G** level of theory in the aqueous phase. ^(A)^ complete double helix and ^(B)^ base-pairs skeleton, NE—non-equilibrated solvent-solute interaction, EQ—equilibrated solvent-solute interaction. The raw data of have been given in Table S2 and Table S3.

System	VIP^NE^	VIP^EQ^	AIP	VEA^NE^	VEA^EQ^	AEA
oligo-RdcG	^(A)^ 6.51	5.89	5.47	−0.99	−1.33	−2.11
	^(B)^ 6.32	5.82	5.40	−0.69	−1.42	−1.90
	Base Pair					
		A_1_T_5_	cG_2_C_4_	A_3_T_3_	^OXO^G_4_C_2_	A_5_T_1_
	VIP	6.69	6.15	6.58	5.93	6.73
	AIP	6.71	6.19	6.54	5.56	6.67
	VEA	−1.38	−1.49	−1.45	−1.53	−1.42
	AEA	−1.38	−1.48	−1.44	−1.97	−1.40
oligo-SdcG	Electronic properties in [eV]					
	^(A)^ 6.55	5.92	5.47	−1.01	−1.60	−2.12
	^(B)^ 6.37	5.86	5.39	−0.68	−1.44	−1.88
	Base Pair					
		A_1_T_5_	cG_2_C_4_	A_3_T_3_	^OXO^G_4_C_2_	A_5_T_1_
	VIP	6.67	6.14	6.64	5.93	6.72
	AIP	6.66	6.18	6.67	5.57	6.68
	VEA	−1.41	−1.55	−1.40	−1.52	−1.42
	AEA	−1.42	−1.94	−1.39	−1.52	−1.42

**Table 3:** Charge transfer parameters. The Δ*G*-driving force, λ-reorganisation energy, *E*_a_-activation energy, *V*_12_-electron coupling, and *k*_HT_-charge rate constant of permissible transfer between base-pairs of oligo-RcdG and oligo-ScdG, calculated at the M06-2X/6-31++G** level of theory in the aqueous phase and given in [eV]. Arrows indicate the direction of charge migration. The raw data of have been given in Table S4a,b.

System	Electron-Hole Transfer				
oligo-RcdG	*λ*	Δ*G*	*E*_a_	*V*_12_	*k*_HT_ (s^−1^)
A_1_T_1_→cG_2_C_2_	−0.04	−0.52	−1.94	0.30	ND
cG_2_C_2_←A_3_T_3_	−0.02	−0.35	−2.04	0.22	ND
A_3_T_3_→^OXO^G_4_C_4_	0.40	−0.99	0.22	0.41	1.04 × 10^12^
^OXO^G_4_C_4_←A_5_T_5_	0.38	−1.11	0.36	0.42	3.83 × 10^9^
A_1_T_1_→A_3_T_3_	0.04	−0.17	0.08	0.05	8.19 × 10^12^
cG_2_C_2_→^OXO^G_4_C_4_	0.39	−0.64	0.04	0.10	5.92 × 10^13^
A_3_T_5_←A_5_T_5_	0.04	−0.13	0.04	0.72	8.36 × 10^13^
oligo-RcdG	Excess Electron Transfer				
A_1_T_1_→cG_2_C_2_	0.00	−0.10	−0.80	0.09	ND
cG_2_C_2_←A_3_T_3_	0.02	−0.05	0.01	0.04	1.22 × 10^14^
A_3_T_3_→^OXO^G_4_C_4_	0.47	−0.54	0.00	0.08	1.46 × 10^14^
^OXO^G_4_C_4_←A_5_T_5_	0.49	−0.57	0.00	0.05	4.74 × 10^13^
A_1_T_1_→A_3_T_3_	−0.02	−0.06	−0.08	0.08	ND
cG_2_C_2_→^OXO^G_4_C_4_	0.45	−0.49	0.001	0.46	5.06 × 10^15^
A_3_T_5_←A_5_T_5_	−0.04	−0.04	−0.04	0.08	ND
System	Electron-Hole Transfer				
oligo-ScdG	*λ*	Δ*G*	*E*_a_	*V*_12_	*k*_HT_(s^−1^)
A_1_T_1_→cG_2_C_2_	−0.04	−0.47	−1.52	0.29	ND
cG_2_C_2_←A_3_T_3_	−0.02	−0.49	−3.48	0.37	ND
A_3_T_3_→^OXO^G_4_C_4_	0.39	−1.11	0.33	0.39	9.39 × 10^9^
^OXO^G _4_C_4_←A_5_T_5_	0.41	−1.11	0.29	0.37	3.96 × 10^10^
A_1_T_1_←A_3_T_3_	−0.02	−0.02	−0.02	0.17	ND
cG_2_C_2_→^OXO^G_4_C_4_	0.39	−0.62	0.03	0.10	7.10 × 10^13^
A_3_T_5_←A_5_T_5_	0.07	−0.001	0.02	0.04	5.80 × 10^13^
oligo-ScdG	Excess Electron Transfer				
A_1_T_1_→cG_2_C_2_	0.50	−0.52	0.00	0.06	7.83 × 10^13^
cG_2_C_2_←A_3_T_3_	0.49	−0.56	0.00	0.04	4.34 × 10^13^
A_3_T_3_→^OXO^G_4_C_4_	0.01	−0.13	0.31	0.11	1.04 × 10^10^
^OXO^G _4_C_4_←A_5_T_5_	0.00	−0.10	7.44	0.05	0.00
A_1_T_1_←A_3_T_3_	0.00	−0.03	−0.56	0.07	ND
cG_2_C_2_←^OXO^G_4_C_4_	0.48	−0.42	0.002	0.05	5.89 × 10^13^
A_3_T_5_→A_5_T_5_	0.02	−0.03	0.01	0.07	5.98 × 10^14^

## Data Availability

Data are contained in the article and Supplementary Materials.

## Supplementary Materials

The following supporting information can be downloaded at: https://www.mdpi.com/article/10.3390/antiox12040881/s1, Table S1 Hirshfeld charge and spin distribution in the shape of oligo-RcdGd [A_1_(5′R)cG_2_A_3_^OXO^G_4_A_5_]*d[T_5_C_4_T_3_C_2_T_1_] and oligo-ScdGd [A_1_(5′S)cG_2_A_3_^OXO^G_4_A_5_]*d[T_5_C_4_T_3_C_2_T_1_], only nucleosides bases were taken into consideration, calculated at the M06-2x/6-31++G** level of theory in the aqueous phase. Vertical Cation (VC^NC^) (NE-non-equilibrated), Vertical Cation (VC^EQ^) (EQ-equilibrated), Vertical Anion (VA^NE^), Vertical Anion (VA^EQ^), Adiabatic Cation (AC), Adiabatic Anion (AA). Table S2 The energies (in Hartree) of Neutral, Vertical Cation, Adiabatic Cation, and Vertical Neutral forms of base pairs extracted from *ds*-oligonucleotides calculated at the M06-2x/6-31++G** level of theory in the aqueous phase. Table S3 The energies (in Hartree) of Neutral, Vertical Cation (VC^NC^) (NE-non-equilibrated), Vertical Cation (VC^EQ^) (EQ-equilibrated), Vertical Anion (VA^NE^), Vertical Anion (VA^EQ^), Adiabatic Cation (AC), Adiabatic Anion (AA) and Vertical Neutral from Cation (VNC^NE^), Vertical Neutral from Cation (VNC^EQ^), Vertical Neutral from Anion (VNA^NE^), Vertical Neutral from Anion (VNA^EQ^) of a complete DNA double helix and base pairs skeleton extracted from *ds*-oligonucleotides, calculated at the M06-2x/6-31+G** and M06-2X/6-31++G** levels of theory in the aqueous phase, respectively. Table S4a. Energies: Ground (E^GR^) and Excitation (E^EX^) state energies and Excitation and HOMO Energies as well as corresponding Dipole Moments Ground, Excitation, and Transition (DM^G^, DM^EX^, D_12_) in Debays of neighbouring base pairs extracted from selected dimers of *ds*-oligonucleotides, calculated at the M06-2x/6-31++G** level of theory in the aqueous phase using the DFT or TD-DFT methodology. Table S4b. Energies: Ground (E^GR^) and Excitation (E^EX^) state energies and Excitation and HOMO Energies as well as corresponding Dipole Moments Ground, Excitation, and Transition (DM^G^, DM^EX^, D_12_) in Debays of a distal base pair extracted from selected trimmers of *ds*-oligonucleotides, calculated at the M06-2x/6-31++G** level of theory in the aqueous phase using the DFT or TD-DFT methodology. The structure of the discussed ds-oligonucleotides is attached as pdf files (cartesian coordinates) in the supplementary materials, as follows: (1) oligo_(R)cdG_NEUTRAL; (2) oligo_(S)cdG_NEUTRAL; (3) oligo_(R)cdG_CATION; (4) oligo_(S)cdG_CATION; (5) oligo_(R)cdG_ANION; (6) oligo_(S)cdG_ANION.
