# Supplementary material for: The Influence of 5′,8-Cyclo-2′-Deoxyguanosine on ds-DNA Charge Transfer Depends on Its Diastereomeric Form: A Theoretical Study
Source: Antioxidants (Basel). 2023 Apr 4;12(4):881. doi: 10.3390/antiox12040881 (PMC10135346; doi:10.3390/antiox12040881)
Supplement: Supplementary file 1 [file antioxidants-12-00881-s001.zip › Suplementary materials Antioxidants.pdf]

Supplementary Materials

The influence of 5',8-cyclo-2'-deoxyGuanosine on ds-DNA charge transfer depends on its diastereomeric form: A theoretical study.

Bolesław Karwowski

Table S1. Hirshfeld charge and spin distribution in the shape of oligo-RcdG $d[A_1(5'R)cG_2A_3{}^{OXO}G_4A_5]*d[T_5C_4T_3C_2T_1]$ and oligo-ScdG $d[A_1(5'S)cG_2A_3{}^{OXO}G_4A_5]*d[T_5C_4T_3C_2T_1]$ only nucleosides bases were taken into consideration, calculated at the M06-2x/6-31++G** level of theory in the aqueous phase. Vertical Cation (VC^{NC}) (NE-non-equilibrated), Vertical Cation (VC^{EQ}) (EQ-equilibrated), Vertical Anion (VA^{NE}), Vertical Anion (VA^{EQ}), Adiabatic Cation (AC), Adiabatic Anion (AA)

**oligo-RcdG**							
	**Neutral**	**VC^{NE}**		**VC^{EQ}**		**AC**	
	**Charge**	**Charge**	**Spin**	**Charge**	**Spin**	**Charge**	**Spin**
**A_1T_5**	-0.08	-0.05	0.00	-0.07	0.00	0.00	-0.06
**(R)cG_2C_4**	-0.09	-0.08	0.00	-0.07	0.00	0.00	-0.07
**A_3T_3**	-0.06	0.09	0.07	0.06	0.09	0.10	0.05
oxo**G_4C_2**	0.10	0.82	0.90	0.87	0.87	0.87	0.84
**A_5T_1**	0.13	0.21	0.03	0.21	0.04	0.03	0.23
		**VA_{NE}**		**VA_{EQ}**		**AA**	
		Charge	Spin	Charge	Spin	Charge	Spin
**A_1T_5**		-0.27	0.15	-0.17	0.07	-0.09	0.00
**(R)cG_2C_4**		-0.78	0.80	-0.36	0.29	-0.09	0.00
**A_3T_3**		-0.14	0.05	-0.24	0.17	-0.14	0.03
oxo**G_4C_2**		0.08	0.00	-0.22	0.33	-0.71	0.94
**A_5T_1**		0.11	0.00	-0.01	0.12	0.03	0.04
**oligo-ScdG**							
	**Neutral**	**VC^{NE}**		**VC^{EQ}**		**AC**	
	**Charge**	**Charge**	**Spin**	**Charge**	**Spin**	**Charge**	**Spin**
**A_1T_5**	-0.13	-0.11	0.00	-0.13	0.00	-0.10	0.00
**(S)cG_2C_4**	-0.02	0.01	0.00	0.00	0.00	-0.01	0.00
**A_3T_3**	-0.08	0.00	0.06	0.00	0.05	0.04	0.05
oxo**G_4C_2**	0.10	0.88	0.91	0.92	0.93	0.86	0.92
**A_5T_1**	0.13	0.22	0.02	0.21	0.02	0.21	0.02
		**VA^{NE}**		**VA^{EQ}**		**AA**	
		Charge	Spin	Charge	Spin	Charge	Spin
**A_1T_5**		-0.39	0.23	-0.47	0.31	-0.28	0.08
**(S)cG_2C_4**		-0.65	0.73	-0.59	0.63	-0.76	0.89
**A_3T_3**		-0.15	0.05	-0.16	0.05	-0.16	0.03
oxo**G_4C_2**		0.08	0.00	0.08	0.01	0.07	0.00
**A_5T_1**		0.11	0.00	0.13	0.00	0.13	0.00

Table S2. The energies (in Hartree) of Neural, Vertical Cation, Adiabatic Cation, and Vertical Neutral forms of base pairs extracted from *ds*-oligonucleotides calculated at the M062x/6-31++G** level of theory in the aqueous phase.

**oligo-RcdG**	**Neutral**	**Vertical Cation**	**Adiabatic Cation**	**Vert Neutral**
**A_1T_5**	-921.192077	-920.946286	-920.945487	-921.191924
**(R)cG_2C_4**	-937.252631	-937.026629	-937.025014	-937.252132
**A_3T_3**	-921.191484	-920.949536	-920.951022	-921.190472
**oxoG_4C_2**	-1012.477664	-1012.25978	-1012.273459	-1012.465991
**A_5T_1**	-921.1921	-920.944765	-920.946948	-921.192204
**oligo-RcdG**		**Vertical Anion**	**Adiabatic Anion**	**Vert Neutral**
**A_1T_5**		-921.242758	-921.242794	-921.192102
**(S)cG_2C_4**		-937.307271	-937.307164	-937.252495
**A_3T_3**		-921.244862	-921.244298	-921.190722
**oxoG_4C_2**		-1012.533766	-1012.55015	-1012.458782
**A_5T_1**		-921.244207	-921.243464	-921.191261
**oligo-ScdG**	**Neutral**	**Vertical Cation**	**Adiabatic Cation**	**Vert Neutral**
**A_1T_5**	-921.19169	-920.94668	-920.947014	-921.191732
**(S)cG_2C_4**	-937.252358	-937.026699	-937.025119	-937.251471
**A_3T_3**	-921.192007	-920.948144	-920.946734	-921.191113
**oxoG_4C_2**	-1012.47807	-1012.260127	-1012.273474	-1012.464719
**A_5T_1**	-921.192101	-920.944996	-920.9468	-921.192205
**oligo-ScdG**		**Vertical Anion**	**Adiabatic Anion**	**Vert Neutral**
**A_1T_5**		-921.243389	-921.243877	-921.190654
**(S)cG_2C_4**		-937.306336	-937.323829	-937.238128
**A_3T_3**		-921.243439	-921.243061	-921.191503
**oxoG_4C_2**		-1012.534077	-1012.534003	-1012.478134
**A_5T_1**		-921.244324	-921.2444	-921.192163

Table S3. The energies (in Hartree) of Neural, Vertical Cation (VC^{NC}) (NE-non-equilibrated), Vertical Cation (VC^{EQ}) (EQ-equilibrated), Vertical Anion (VA^{NE}), Vertical Anion (VA^{EQ}), Adiabatic Cation (AC), Adiabatic Anion (AA) and Vertical Neutral from Cation (VNC^{NE}), Vertical Neutral from Cation (VNC^{EQ}), Vertical Neutral from Anion (VNA^{NE}), Vertical Neutral from Anion (VNA^{EQ}) of complete DNA double helix and base pairs skeleton extracted from *ds*-oligonucleotides calculated at the M06-2x/6-31+G** and M06-2X/6-31++G** level of theory in the aqueous phase, respectively.

	**Neutral**	**VC^{NE}**	**VC^{EQ}**	**VA^{NE}**	**VA^{EQ}**	**AC**	**AA**	**VNC^{NE}**	**VNC^{EQ}**	**VNA^{NE}**	**VNA^{EQ}**
	**Complete DNA double helix**										
**oligo-RcdG**	-12850,3473062	-12850,1082927	-12850,1308891	-12850,3838106	-12850,3962294	-12850,1463089	-12850,4246506	-12850,3301861	-12850,3301861	-12850,3260601	-12850,3260601
**oligo-ScdG**	-12850,3684644	-12850,1507938	-12850,1507938	-12850,4272361	-12850,4272361	-12850,1676202	-12850,4462867	-12850,3524415	-12850,3524415	-12850,3500974	-12850,3503722
**Base Pairs skeleton extracted from *ds*-oligonucleotides**											
**oligo-RcdG**	-4864,9857110	-4864,7459109	-4864,7687890	-4865,0096060	-4865,0359660	-4864,7837180	-4865,0539290	-4864,9633460	-4864,9706250	-4864,9609280	-4864,9667280
**oligo-ScdG**	-4713,4003200	-4713,1662740	-4713,1851550	-4713,4253210	-4713,4530600	-4713,2021870	-4713,4694840	-4713,3738530	-4713,3847740	-4713,3775870	-4713,3817270

Table S4a. The Energies: Ground (E^{GR}) and Excitation (E^{EX}) state energies and Excitation and HOMO Energies as well as corresponding Dipole Moments Ground, Excitation, and Transition (DM^{G}, DM^{EX}, D_{12}) in Debays of neighbor base pair extracted from selected dimmers of *ds*oligonucleotides, calculated at the M06-2x/6-31++G** level of theory in the aqueous phase using the DFT or TD-DFT methodology.

SYSTEM	B.P. Dimer	E_{GR}	DM^{GR}	E_{EX}	DM^{EX}	D_{12}	E_{HOMO}	E_{HOMO-1}	E_{LUMO}	E_{LUMO+1}
**oligo-RcdG**	**A_1|| (R)cG_2**	-1858.463603	9.33	-1858.329977	9.60	11.80	-0.2616	-0.2833	-0.0182	-0.0117
	**(R)cG_2||A_3**	-1858.463822	10.22	-1858.329757	10.27	11.77	-0.2626	-0.2788	-0.0163	-0.0134
	**A_3||$_{oxo}G_4$**	-1933.694083	16.86	-1933.569293	15.11	4.05	-0.2521	-0.2832	-0.0182	-0.0121
	**$_{oxo}G_4$||A_5**	-1933.695588	16.01	-1933.570371	14.19	7.33	-0.2534	-0.2842	-0.0182	-0.0147
**oligo-ScdG**	**A_1|| (S)cG_2**	-1858.464337	9.50	-1858.331441	9.77	7.05	-0.2592	-0.2805	-0.0198	-0.0099
	**(S)cG_2||A_3**	-1858.466173	8.89	-1858.33217	8.90	12.77	-0.2611	-0.2791	-0.0160	-0.0127
	**A_3||$_{oxo}G_4$**	-1933.693736	16.20	-1933.567051	14.39	6.09	-0.2541	-0.2818	-0.0182	-0.0097
	**$_{oxo}G_4$||A_5**	-1933.695372	16.28	-1933.568529	14.56	4.26	-0.2543	-0.2836	-0.0177	-0.0141

Table S4b. The Energies: Ground (E^{GR}) and Excitation (E^{EX}) state energies and Excitation and HOMO Energies as well as corresponding Dipole Moments Ground, Excitation, and Transition (DM^{G}, DM^{EX}, D_{12}) in Debays of distal base pair extracted from selected trimmers of *ds*oligonucleotides, calculated at the M06-2x/6-31++G** level of theory in the aqueous phase using the DFT or TD-DFT methodology

**SYSTEM**	**Base Pair Dimer**	**E_{GR}**	**DM^{GR}**	**E_{EX}**	**DM^{EX}**	**D_{12}**	**E_{HOMO}**	**E_{HOMO-1}**	**E_{LUMO}**	**E_{LUMO+1}**
**oligo-RcdG**	**A_1||A_3**	-1858.463603	9.33	-1858.329977	9.60	11.80	-0.261585	-0.283314	-0.018154	-0.01174
	**(R)cG_2||G_4**	-1858.463822	10.22	-1858.463822	10.27	11.77	-0.262626	-0.278803	-0.016321	-0.013445
	**A_3||A_5**	-1933.694083	16.86	-1933.694083	15.11	4.05	-0.252082	-0.283172	-0.018217	-0.012126
**oligo-ScdG**	**A_1||A_3**	-1858.464337	9.50	-1858.331441	9.77	**7.05**	-0.259245	-0.280511	-0.019773	-0.009899
	**(S)cG_2||G_4**	-1858.466173	8.89	-1858.33217	8.90	**12.77**	-0.2611	-0.279117	-0.015991	-0.012727
	**A_3||A_5**	-1933.693736	16.20	-1933.567051	14.39	**6.09**	-0.254119	-0.281835	-0.01822	-0.009693
